# Taurine in canine physiology and metabolic regulation

**DOI:** 10.3389/fvets.2026.1812065

**Published:** 2026-07-22

**Authors:** Pengpeng Wang, Min Yang, Yuxiang Yang, Fuxiao Xiu, Chengwu Liu, Shibing Zhou

**Affiliations:** Police Dog Technology Department, Criminal Investigation Police University of China, Shenyang, China

**Keywords:** canine, molecular mechanism, nutritional deficiency, physiological function, precision nutrition, taurine

## Abstract

Taurine is a conditionally essential amino acid in dogs and plays a fundamental role in maintaining physiological homeostasis and metabolic health. This review systematically summarizes the biosynthesis, tissue distribution, and core physiological functions of taurine in dogs, and provides an in-depth analysis of its regulatory mechanisms across multiple organ systems, including the myocardium, retina, nervous system, reproductive system, and lipid metabolism. Particular emphasis is placed on the mechanistic links between taurine deficiency and dilated cardiomyopathy, a prevalent and clinically significant cardiac disorder in dogs. In addition, major determinants influencing taurine metabolic homeostasis are examined, with particular attention to genetic variability among breeds, differences related to body size, changes across physiological stages, and the modulatory effects of dietary composition. Accumulating evidence indicates substantial inter-individual variability in endogenous taurine synthesis capacity in dogs, which is generally insufficient to meet dynamic physiological demands under various conditions. Consequently, exogenous dietary supplementation has emerged as a critical strategy for maintaining taurine homeostasis and supporting canine health. By integrating current advances in taurine-related canine nutrition and physiology, this review provides a scientific framework for the development of precision nutritional strategies, the prevention and management of metabolic disorders, and the formulation of functional dog foods, while highlighting key knowledge gaps and future research directions in this field.

## 1 Introduction

With the increasingly prominent roles of companion and working dogs in human society, growing attention has been directed toward their health management and nutritional science. Taurine, a non-proteinogenic amino acid with unique biological activity, has become an indispensable functional nutrient in canine diets owing to its diverse physiological regulatory properties. Although the fundamental physiological functions of taurine have been extensively investigated in mammals, substantial knowledge gaps remain regarding dogs as a distinct species. In particular, the species-specific regulatory mechanisms of taurine, the dynamic nutritional requirements influenced by breed-related genetic background, body size, and physiological stages (e.g., growth, pregnancy, and aging), as well as the in-depth association with common canine metabolic diseases (e.g., dilated cardiomyopathy and lipid metabolism disorders), still have many scientific issues to be clarified.

Existing evidence indicates that taurine deficiency is closely associated with the development of canine dilated cardiomyopathy (DCM), with certain dog breeds exhibiting pronounced genetic susceptibility (1–4). In addition, findings from studies in various mammalian species suggest that taurine plays critical regulatory roles in maintaining retinal function, modulating neuro-behavior, supporting reproductive health, and regulating lipid metabolism; however, direct evidence in dogs remains limited for several of these functions (5–8).

Against this background, the present review systematically integrates current knowledge on the biosynthesis and transport mechanisms of taurine, as well as its core physiological functions in dogs. Furthermore, it provides an in-depth analysis of taurine-mediated regulatory pathways and underlying molecular mechanisms across multiple organ systems, drawing on mechanistic insights from mammalian studies while explicitly distinguishing evidence derived from dogs from extrapolations based on other species. Collectively, this review aims to provide a comprehensive scientific basis to support the development of individualized nutritional strategies, facilitate the early prevention and management of metabolic diseases, promote the formulation of functional pet foods, and advance canine nutritional science toward precision- and prevention-oriented approaches.

## 2 Biosynthesis, tissue distribution, and core physiological functions of taurine

Taurine (2-aminoethanesulfonic acid) is one of the most abundant free amino acids in canine tissues, with exceptionally high concentrations in the myocardium, retina, and brain. Among the four principal sulfur-containing amino acids in mammals—methionine, cysteine, homocysteine, and taurine—only methionine and cysteine are incorporated into proteins. Endogenous taurine synthesis in dogs, as in most mammals, relies on methionine and cysteine as precursor substrates, proceeding through the sequential catalytic actions of key enzymes including cysteine dioxygenase (CDO) and cysteine sulfinate decarboxylase (CSD) (9–11). Of these two primary precursor amino acids, methionine is indispensable for dogs and must be supplied through dietary intake, whereas cysteine can be synthesized endogenously from methionine via the transsulfuration pathway. This metabolic route proceeds through a series of intermediates—S-adenosylmethionine (SAM), S-adenosylhomocysteine (SAH), and homocysteine—before ultimately yielding cysteine in an irreversible manner (12). This irreversibility carries critical physiological implications: when dietary methionine supply is restricted, the synthesis of downstream cysteine is directly constrained, thereby limiting multiple metabolic processes including taurine biosynthesis. Cysteine serves as the immediate precursor for taurine, and their conversion is accomplished through a three-enzyme cascade that constitutes a complete linear metabolic pathway. Specifically, cysteine is first oxidized to cysteine sulfinate by CDO, then decarboxylated to hypotaurine by CSD, and finally oxidized to taurine by hypotaurine dehydrogenase (HTAU-DH). This sequential pathway exhibits a strict substrate dependency: the efficiency of each downstream reaction relies on an adequate supply of the upstream substrate (12).

This biosynthetic framework carries two interrelated nutritional implications for dogs. On one hand, when dietary intake of methionine and cysteine—collectively designated as sulfur amino acids (SAA)—is insufficient, the precursor pool for taurine synthesis becomes depleted, leading directly to diminished endogenous taurine production. This concern is particularly pronounced in dogs consuming plant protein-based diets, which are inherently lower in SAA compared with animal-derived proteins. On the other hand, inherent metabolic differences exist across breeds: certain dog breeds exhibit lower hepatic activities of taurine biosynthetic enzymes, such that even when dietary SAA intake meets established minimum nutritional requirements, the efficiency of taurine conversion may remain suboptimal. In summary, taurine nutritional status in dogs cannot be considered in isolation; rather, it is tightly coupled with the metabolic status of methionine and cysteine. These three sulfur-containing amino acids function as an integrated metabolic regulatory system, with precursor availability serving as the primary determinant of taurine synthetic capacity. Beyond this precursor dependency, individual factors including body size, age, and breed also significantly influence endogenous taurine biosynthetic capacity. Large-breed dogs exhibit markedly lower rates of taurine synthesis compared with small-breed dogs (13), while the activity of key taurine biosynthetic enzymes is relatively high during early life but progressively declines with aging, substantially reducing synthesis capacity in older dogs (14). These inherent limitations in endogenous production indicate that, for the majority of dog breeds, *de novo* synthesis alone is insufficient to meet physiological taurine demands across all life stages, rendering exogenous dietary supplementation a critical strategy for maintaining taurine homeostasis.

The tissue distribution of taurine in dogs is highly specific, with preferential enrichment in electrically excitable tissues and metabolically active organs. Although comprehensive mapping of taurine distribution across all canine tissues is limited, studies in other mammalian species, such as hamsters, have shown that taurine concentrations are particularly high in the retina, myocardium, skeletal muscle, brain, and liver, with relatively high levels also observed in the kidneys and reproductive organs (15). In dogs, a recent study comparing taurine levels in blood and cardiac tissue across breeds provides direct evidence supporting the relevance of this distribution pattern to canine physiology (16). This distinct distribution pattern is primarily dependent on the active transport function of the sodium- and chloride-dependent taurine transporter (TauT/SLC6A6), which facilitates efficient transmembrane uptake of taurine. As a result, intracellular taurine concentrations can reach levels several 100-fold higher than those in the extracellular environment, thereby establishing a stable concentration gradient that is essential for taurine’s physiological actions. For instance, the exceptionally high taurine content in the retina is closely associated with its antioxidant capacity and its critical role in maintaining photoreceptor integrity and function, whereas taurine enrichment in cardiomyocytes is fundamental to the preservation of myocardial contractile performance and cardiac energy metabolism (5, 17).

The physiological functions of taurine are pleiotropic and fundamental, constituting a shared mechanistic basis for its regulatory roles across multiple organ systems. As a key organic osmolyte—though not the sole one—taurine contributes to maintaining normal cell volume and morphology in tissues such as the brain and kidneys by regulating intracellular and extracellular osmotic balance, thereby protecting cells from osmotic stress-induced damage (18–20). In addition, taurine plays a central role in calcium signaling by modulating the activity of sarcoplasmic reticulum Ca^2+^ pumps and enhancing myofibrillar sensitivity to Ca^2+^, thereby contributing to essential physiological processes such as myocardial contraction and neuronal signal transmission and serving as a critical determinant of excitation–contraction coupling (21). Taurine also exerts potent antioxidant and cytoprotective effects by directly scavenging reactive oxygen species, including hypochlorous acid (HOCl) generated by neutrophils, to form the less toxic taurine chloramine, while simultaneously preserving the activity of endogenous antioxidant enzymes such as superoxide dismutase and catalase, ultimately attenuating oxidative stress–induced cellular injury (22, 23).

**Figure 1:**
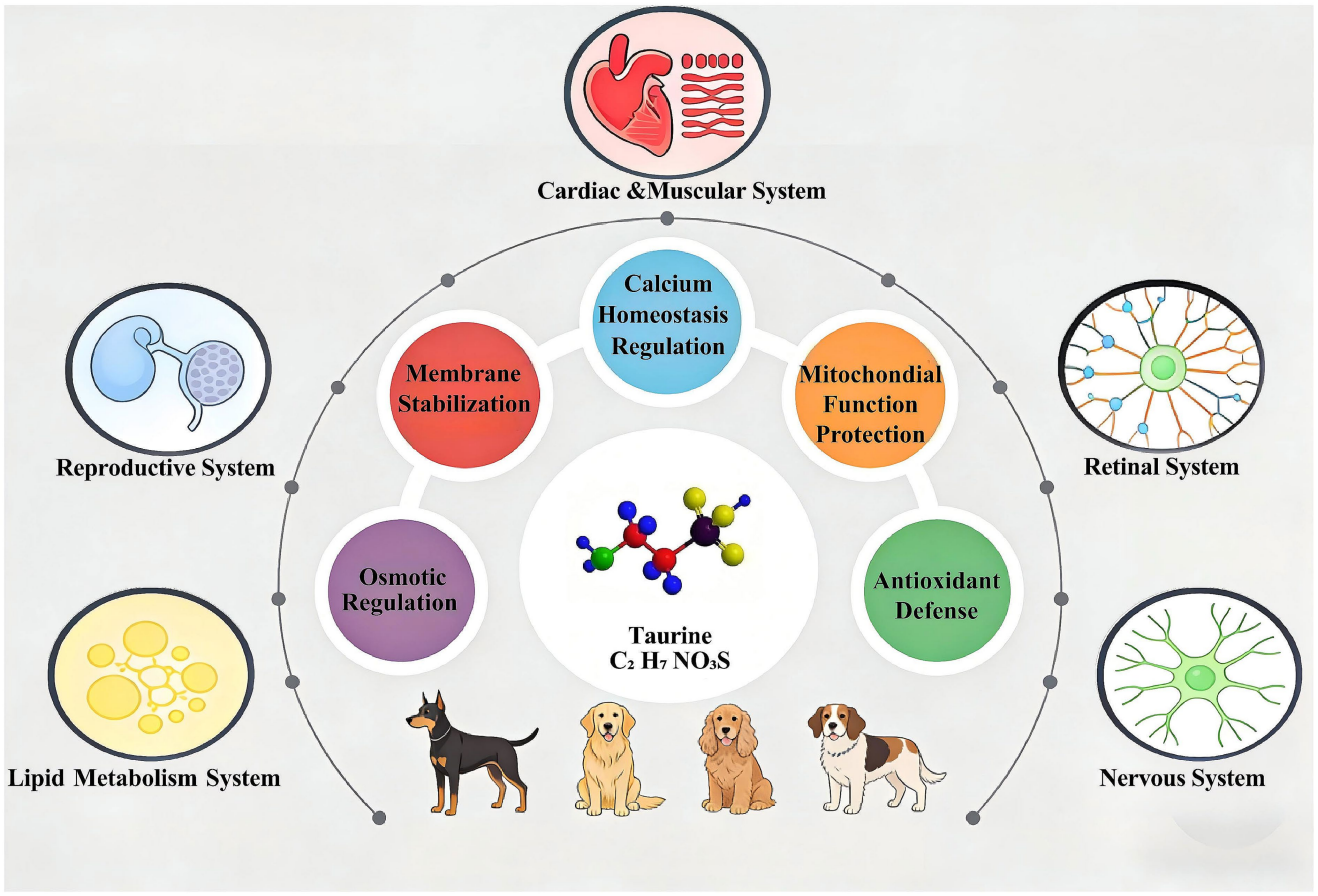
Taurine-mediated multi-organ regulatory network in canine physiological homeostasis and nutritional regulation.

Furthermore, as an essential component of conjugated bile acids (e.g., taurocholic acid), taurine participates in the emulsification, digestion and absorption of dietary lipids and contributes to lipid metabolic homeostasis. This aspect of taurine function is particularly critical in dogs, as they conjugate bile acids almost exclusively with taurine, unlike many other species that can also utilize glycine for conjugation. Consequently, dogs experience continuous taurine loss through biliary secretion, and factors that increase fecal bile acid excretion—such as dietary soluble fiber—can significantly impact taurine homeostasis (24, 25). Through electrostatic interactions with membrane phospholipids, taurine additionally modulates phospholipid composition (e.g., the phosphatidylethanolamine/phosphatidylcholine (PE/PC) ratio), thereby maintaining membrane fluidity and integrity and ensuring the proper function of membrane-bound enzymes and transporters (26). Collectively, these interconnected physiological functions enable taurine to form a complex regulatory network in dogs, providing integrated support for the maintenance of normal physiological functions across diverse organ systems (Figure 1). However, it is important to note that much of the mechanistic understanding of these processes is derived from studies in laboratory animals and other mammalian species; direct experimental confirmation in dogs remains limited for several of these pathways.

## 3 Regulatory roles and mechanisms of taurine in various canine organ systems

### 3.1 Cardiac and muscular systems

Accurate assessment of taurine status is a prerequisite for investigating its relationship with DCM in dogs. Among the three commonly used biological matrices—whole blood, plasma, and serum—whole blood taurine concentration (WB-TauC) is considered the most reliable indicator of long-term taurine homeostasis, as it remains stable during physiological perturbations such as short-term fasting. In contrast, heparinized plasma taurine concentration (Hep-TauC) reflects a more dynamic circulating taurine pool (27). In terms of sample type selection, serum is not recommended, because the clotting process triggers taurine release from platelets, leading to falsely elevated measurements; heparinized plasma is therefore the preferred specimen for clinical measurement.

For the interpretation of results, the most widely used criteria are those established based on extensive clinical experience at the UC Davis Veterinary Medicine Amino Acid Laboratory, with foundational quantitative data provided by Delaney et al. (28, 29). These thresholds define reduced taurine status as WB-TauC below 200 nmol/mL or Hep-TauC below 60 nmol/mL, and critically low levels as WB-TauC below 150 nmol/mL or Hep-TauC below 40 nmol/mL (14). Of note, subsequent reference interval studies conducted across different breed populations have revealed that analytical platforms (e.g., HPLC versus LC–MS), sample processing protocols, and breed composition all contribute to variations in the upper and lower limits of reference ranges (Table 1). Consequently, clinical decision-making should not rely on a single fixed cutoff; rather, it should integrate laboratory-specific reference intervals, breed susceptibility, and dietary history. For breeds with a known genetic predisposition, maintaining taurine concentrations in the upper half of the reference range represents a prudent clinical strategy.

**Table 1:** Summary of reference data and breed-specific taurine concentrations in canine whole blood, plasma, and serum.

Study/Source	Population	Matrix	Taurine concentration (nmol/mL)	Key remarks
Delaney et al. (28)	131 dogs (multi-breed)	Whole blood	266 ± 5.1 (mean ± SE)	Foundational dataset; significant inter-breed differences
		Heparinized plasma	77 ± 2.1 (mean ± SE)	
	Australian shepherds	Whole blood	222 ± 10.2	inter-breed comparison
	Labrador retrievers	Whole blood	294.6 ± 14.1	Significantly higher than Australian shepherds (*p* = 0.0003)
Kriström et al. (14)	—	Whole blood	<200 (reduced); <150 (critically low)	Derived from Delaney et al. (28) and refined with clinical experience
		Heparinized plasma	<60 (reduced); <40 (critically low)	
Ontiveros et al. (99)	43 golden retrievers (normal echocardiography)	Whole blood	213–377 (reference interval)	Breed-specific reference interval; UPLC–MS/MS method
		Heparinized plasma	63–194 (reference interval)	
Furlanello et al., (100)	120 dogs (41 breeds)	Whole blood	148–495 (reference interval)	Multi-breed reference interval; validated LC–MS method
		Heparinized plasma	42–183 (reference interval)	
Kathrani et al. (101)	12 healthy control dogs	Serum	110–272 (range)	Healthy control serum range; HPLC method

SE, standard error; HPLC, high-performance liquid chromatography; LC–MS, liquid chromatography–tandem mass spectrometry; UPLC-MS/MS, ultra-performance liquid chromatography–tandem mass spectrometry.

Within this assessment framework, the regulatory role of taurine in the cardiovascular system has emerged as a major research focus, with its clinical significance underscored by the well-established link between taurine deficiency and DCM. As the second most prevalent cardiac disease in dogs, DCM exhibits a particularly high incidence in certain breeds, with prevalence exceeding 50% in Doberman Pinschers, while American Cocker Spaniels, Golden Retrievers, and Newfoundlands also demonstrate marked disease susceptibility (30, 31). It is important to distinguish between two forms of DCM in dogs: genetic (primary) DCM, which is associated with breed-specific predispositions and often presents with low blood taurine levels, and diet-associated DCM, which can occur in breeds not typically considered susceptible and may develop with normal or only mildly reduced taurine levels (32). Accumulating evidence indicates that taurine deficiency represents a critical risk factor for the development of nutritional DCM in dogs, and that taurine supplementation can significantly improve—and in some cases reverse—cardiac dysfunction in affected individuals (32).

The relationship between taurine status and DCM has been extensively documented in the veterinary literature. Foundational studies established that taurine deficiency can lead to reversible cardiomyopathy in dogs, with breed susceptibility playing a key role (1–4). In American Cocker Spaniels, the Multicenter Spaniel Trial (MUST) demonstrated that taurine and carnitine supplementation improved cardiac function in affected dogs with decreased plasma taurine concentrations (33). Similarly, Golden Retrievers fed certain commercial diets have been shown to develop taurine deficiency and DCM, with dietary modification and supplementation leading to clinical improvement (34). These findings highlight that DCM in dogs can arise from both genetic predispositions and dietary factors, with taurine deficiency serving as a common pathogenic mechanism in a substantial subset of cases (35).

Taurine plays a pivotal role in maintaining myocardial calcium homeostasis through multiple complementary mechanisms, thereby ensuring efficient excitation–contraction coupling. At physiological concentrations, taurine enhances myofibrillar sensitivity to Ca^2+^, promotes Ca^2+^ release from and reuptake into the sarcoplasmic reticulum (SR), and optimizes myocardial contractile and relaxation performance (36). These mechanistic insights are derived primarily from studies in other species, including rats and fish, and are considered applicable to canine cardiac physiology given the conserved nature of excitation-contraction coupling across vertebrates. In contrast, taurine deficiency leads to profound impairments in cardiomyocyte calcium handling. Increased phosphorylation of troponin I interferes with Ca^2+^ binding to troponin C, resulting in diminished ventricular pressure generation (37). Concurrently, reduced activity of SR calcium pumps disrupts Ca^2+^ reuptake, leading to decreased myocardial contractility, impaired diastolic function, and ultimately heart failure (38). Experimental evidence from taurine transporter knockout mice and taurine-deficient feline models further confirms that dysregulated calcium homeostasis represents a core mechanism underlying taurine deficiency–induced myocardial dysfunction (37, 38).

Given the exceptionally high metabolic demands of cardiomyocytes, mitochondrial integrity is essential for normal cardiac function. Studies in mice and cell culture systems have demonstrated that taurine contributes to myocardial energy metabolic homeostasis by regulating mitochondrial tRNA modification and respiratory chain activity. Specifically, taurine is required for the formation of 5-taurinomethyluridine (τm^5^U), a critical modification at the wobble position of mitochondrial tRNA that is indispensable for efficient translation of mitochondrially encoded respiratory chain complex proteins (39). Taurine deficiency results in reduced τm^5^U synthesis, impaired assembly and function of respiratory chain complexes, diminished electron transport efficiency, inadequate ATP production, and excessive generation of reactive oxygen species (ROS) (39, 40). Moreover, taurine deficiency inhibits long-chain fatty acid uptake by cardiomyocytes, leading to insufficient substrate availability for oxidative metabolism and further exacerbating myocardial energy deficits (17, 41). This assessment framework is particularly relevant for evaluating cardiac patients, given that taurine deficiency has been most extensively studied in the context of DCM.

Beyond taurine, carnitine is another nutritionally significant molecule in canine myocardial energy metabolism. Carnitine transports long-chain fatty acids into the mitochondrial matrix for *β*-oxidation, a process critical for cardiac ATP production (16). Deficiencies in either taurine or carnitine have each been independently linked to the pathogenesis of canine DCM. In American Cocker Spaniels presenting with DCM and markedly low plasma taurine concentrations (mean 15 ± 17 nmol/mL), combined supplementation with both nutrients produced significant improvements in echocardiographic parameters and allowed for successful discontinuation of cardiovascular medications (33). Although direct molecular-level evidence for a synergistic interaction between taurine and carnitine remains limited, their distinct yet complementary functions—taurine preserves mitochondrial integrity and calcium homeostasis (38), while carnitine supports fatty acid oxidation—suggest that concurrent deficiencies may exert additive or even synergistic detrimental effects on myocardial energy metabolism. This observation is particularly relevant for breeds with known genetic predispositions to disorders of taurine or carnitine metabolism, and underscores the need for further investigation via well-controlled studies in dogs.

Under pathological or mechanical stress, myocardial tissue is particularly susceptible to oxidative injury. Taurine mitigates oxidative stress–induced cardiomyocyte damage by directly scavenging hypochlorous acid and suppressing mitochondrial ROS production (42). In parallel, taurine modulates myocardial membrane phospholipid composition by inhibiting phosphatidylethanolamine N-methyltransferase activity, thereby preserving membrane fluidity and structural integrity. This stabilization ensures the proper function of membrane-bound transporters, including Ca^2+^ pumps, and enhances cardiomyocyte resilience to both oxidative and mechanical stress (43). In canine models of heart failure, taurine supplementation has been shown to suppress excessive activation of the renin–angiotensin–aldosterone system (RAAS), reduce angiotensin II levels, decrease cardiac afterload, improve cardiac performance, and prolong survival (44). Notably, similar cardio-protective effects have been reported in human heart failure patients, where short-term taurine supplementation significantly enhances exercise tolerance, reduces myocardial oxygen consumption, and improves cardiac electrical stability (45).

Beyond the myocardium, taurine plays a critical regulatory role in canine skeletal muscle growth, functional maintenance, and exercise endurance. Skeletal muscle from taurine transporter knockout mice exhibits severe functional impairment, characterized by reduced muscle strength and diminished exercise capacity (46). In dogs, exercise stress is associated with a marked decline in skeletal muscle taurine content, whereas exogenous taurine supplementation enhances excitation–contraction coupling by improving SR Ca^2+^ storage and release capacity. Concurrently, taurine attenuates exercise-induced oxidative stress and myofiber damage, thereby improving overall exercise performance and endurance. Specifically, studies in rats have demonstrated that taurine administration modulates skeletal muscle function through calcium-dependent mechanisms (47–49), while investigations in dogs have shown that exercise stress reduces skeletal muscle taurine content and that supplementation may improve performance (50). These effects are mediated through mechanisms analogous to those observed in cardiac muscle, involving the coordinated regulation of calcium homeostasis, antioxidant defense, and membrane stabilization.

### 3.2 Regulation of retinal function

Taurine is one of the most abundant free amino acids in the canine retina, with concentrations markedly exceeding those observed in other tissues. This enrichment is closely associated with the unique physiological characteristics of the retina, including high metabolic activity, elevated oxygen consumption, and prolonged exposure to photo-oxidative stress (5). The survival and functional integrity of retinal cells are highly dependent on the maintenance of redox homeostasis, as excessive accumulation of ROS can induce photoreceptor degeneration, retinal ganglion cell injury, and ultimately visual dysfunction (51–53). Clinical investigations have demonstrated that dogs with primary glaucoma (PG) exhibit significantly reduced levels of taurine and glutamate in damaged photoreceptor regions, indicating a strong association between taurine deficiency and retinal structural degeneration (54).

The retinal protective effects of taurine are mediated through multiple synergistic mechanisms, with evidence derived from studies in various mammalian species, including rats and cattle (55–57). First, the amino group of taurine rapidly reacts with HOCl produced by activated neutrophils to form taurine chloramine, a compound with lower cytotoxicity and anti-inflammatory properties, thereby attenuating oxidative damage to retinal cells (55). Second, taurine preserves mitochondrial respiratory chain integrity, reducing mitochondrial superoxide production and limiting ROS accumulation at its source—an effect that is particularly critical for photoreceptors with exceptionally high metabolic demands (56). In addition, taurine stabilizes the conformational structure of key antioxidant enzymes, including superoxide dismutase and catalase, preventing their oxidative inactivation and enhancing the overall antioxidant capacity of retinal tissue (56). Notably, studies in rodent models of retinal degeneration have shown that taurine deficiency directly promotes photoreceptor degeneration and retinal ganglion cell apoptosis, whereas taurine supplementation delays the progression of retinal degenerative disorders by suppressing apoptotic signaling pathways and maintaining intracellular homeostasis (57). While direct mechanistic studies in dogs are limited, these conserved pathways are likely relevant to canine retinal health, and the observed association between taurine deficiency and retinal damage in dogs with glaucoma supports this extrapolation (54). Collectively, these mechanisms underscore the pivotal role of taurine in the prevention and management of canine retinal diseases, particularly in mitigating age-related retinal degeneration and photo-oxidative stress-induced visual decline.

### 3.3 Regulation of the nervous system

Taurine plays a pivotal neuro-modulatory role in the canine central nervous system, with its core mechanism attributable to its function as an endogenous agonist of the *γ*-aminobutyric acid type A receptor (GABA-AR). By mimicking the inhibitory neurotransmitter GABA, taurine promotes chloride ion influx, suppresses neuronal excitability, and maintains the balance between excitation and inhibition within the brain (6, 58–60). This mechanistic understanding is derived primarily from studies in rodents and cell culture systems; direct experimental evidence in dogs remains limited. However, given the conserved nature of GABAergic signaling across mammals, these findings are considered relevant to canine neurophysiology. This fundamental mechanism underlies taurine’s diverse roles in neuroprotection, emotional regulation, and the maintenance of cognitive function.

In a mouse model of developmental lead exposure, lead-induced disruption of GABAergic system development resulted in anxiety-like behaviors and impaired frontal executive function in adulthood. Supplementation with taurine and its derivatives significantly ameliorated these behavioral and cognitive deficits by restoring GABA receptor function and suppressing neuro-inflammation, with the observed neuroprotective effects exhibiting sex-dependent differences (61). Similarly, in a mouse model of depression induced by chronic social defeat stress, extracellular taurine levels were markedly reduced in emotion-regulating brain regions such as the medial prefrontal cortex. Exogenous taurine administration effectively reversed depression-like behaviors by preserving dendritic spine architecture and promoting the expression of synaptic proteins, including N-methyl-D-aspartate (NMDA) receptors, highlighting a mechanism closely linked to the maintenance of synaptic plasticity (62).

Anxiety-related disorders—including generalized anxiety, separation anxiety, and noise phobia—are prevalent behavioral problems in dogs (63). A large-scale Finnish survey involving 13,700 companion dogs reported that over 70% exhibited at least one anxiety-related behavior, with approximately 32% displaying noise sensitivity (64). Accumulating evidence from rodent models suggests that taurine may influence emotional regulation through modulation of GABAergic signaling; however, direct evidence in dogs remains limited. Extrapolating from these mechanistic insights, it is hypothesized that taurine supplementation could enhance emotional stability and attenuate anxiety-like behaviors in dogs, though controlled canine studies are needed to confirm this effect (65). For working dogs, such as police and search-and-rescue dogs exposed to prolonged high-intensity operational demands, adequate taurine may contribute to the maintenance of cognitive performance, emotional resilience, and sustained work capacity, underscoring its potential importance in the optimization of nutritional strategies for working canines.

Beyond its neuro-modulatory effects, taurine also functions as a key neurotrophic factor involved in the long-term maintenance of brain development and cognitive function in dogs (65). Taurine supports cognitive integrity by promoting neuronal survival, enhancing synaptic transmission efficiency, and mitigating oxidative stress–induced neural damage. Although direct experimental evidence in dogs remains limited, findings from other mammalian models, together with clinical observations in canines, suggest that taurine holds considerable potential in the prevention of age-related cognitive dysfunction and in the improvement of learning and memory performance in elderly dogs.

### 3.4 Regulation of the reproductive system

Taurine is widely distributed throughout the canine reproductive system and exerts important regulatory effects on reproductive function. Although direct experimental evidence in dogs remains relatively limited, conserved mechanisms across mammalian species, together with findings from canine-assisted reproductive technologies, have provided initial confirmation of taurine’s critical role in reproductive health (7, 66).

High concentrations of taurine are detected in the testes, epididymides, and semen. Taurine in male reproductive tissues is derived from both local synthesis via the CSD pathway, as demonstrated in rat studies (9), and systemic transport mediated by the taurine transporter TauT/SLC6A6, as shown in mouse models (10). Studies in rats have shown that taurine contributes to the regulation of the hypothalamic–pituitary-testicular (HPT) axis by promoting luteinizing hormone (LH) secretion, thereby enhancing testosterone biosynthesis in Leydig cells and providing a hormonal milieu conducive to spermatogenesis (7). At the cellular level, evidence from rodent studies indicates that taurine supports spermatogenic progression by enhancing autophagic activity and suppressing apoptotic signaling pathways, which together preserve the structural integrity of the seminiferous epithelium (67). Taurine enrichment within the epididymis further facilitates the acquisition of sperm motility and the maintenance of sperm membrane stability. Taurine deficiency in rodent models is associated with reduced sperm motility and abnormal acrosome reactions, whereas *in vitro* taurine supplementation significantly improves sperm kinematic parameters and fertilization capacity (68, 69). In addition, taurine exerts potent antioxidant effects within the male reproductive tract by scavenging ROS, elevating glutathione (GSH) levels and antioxidant enzyme activities, reducing lipid peroxidation products such as malondialdehyde (MDA), and protecting sperm membranes from oxidative injury (70, 71).

In canine-assisted reproductive technologies, taurine has been widely incorporated into optimized sperm cryopreservation media. During the freeze–thaw process, spermatozoa are highly susceptible to oxidative stress, membrane lipid reorganization, and dysregulated Ca^2+^ influx. A study in dogs demonstrated that taurine supplementation significantly improves post-thaw sperm motility, membrane integrity, and acrosomal integrity through its combined antioxidant, membrane-stabilizing, and calcium homeostasis–modulating properties (66, 72).

Taurine is also present at relatively high concentrations in canine uterine fluid and the oviduct, where it is thought to participate in the regulation of oocyte maturation, facilitation of fertilization, and support of early embryonic development. Studies in other mammalian species indicate that taurine contributes to the establishment of a favorable reproductive microenvironment by regulating oviductal osmotic balance and suppressing oxidative stress, thereby promoting oocyte competence and embryo transport (11, 73). Moreover, studies in rodent models have demonstrated protective effects of taurine against reproductive dysfunction induced by environmental toxicants (e.g., heavy metals and pesticides) (74), highlighting its potential relevance to preserving the developmental and functional integrity of the canine reproductive system, though direct evidence in dogs is lacking.

### 3.5 Regulation of lipid metabolism

Taurine plays a pivotal role in maintaining lipid metabolic homeostasis through an integrated, multi-target regulatory network that encompasses lipid digestion and absorption, lipid synthesis and catabolism, and the modulation of insulin sensitivity. As discussed in Section 2, dogs conjugate bile acids almost exclusively with taurine, making taurine homeostasis particularly sensitive to alterations in bile acid metabolism. This unique aspect of canine physiology underscores the importance of taurine in lipid digestion and absorption, as taurine-conjugated bile acids are essential for the emulsification of dietary lipids (75).

By promoting cholesterol conversion to bile acids via upregulation of cholesterol 7α-hydroxylase (CYP7A1) and enhancing hepatic low-density lipoprotein receptor (LDLR) activity, taurine effectively lowers circulating cholesterol levels (75, 76). Experimental studies in human hepatoma HepG2 cells have demonstrated that taurine induces CYP7A1 mRNA expression, thereby accelerating cholesterol catabolism and biliary excretion (77). Consistently, studies in rodents fed high-cholesterol diets have shown that taurine supplementation reduces serum total cholesterol levels and hepatic cholesterol ester secretion, ultimately attenuating hepatic lipid accumulation (78, 79).

Taurine further suppresses lipid synthesis while promoting lipid catabolism through activation of the AMP-activated protein kinase (AMPK) signaling pathway, as demonstrated in mouse models (80). AMPK activation subsequently stimulates peroxisome proliferator-activated receptor *γ* coactivator 1*α* (PGC1α), facilitating the browning of white adipose tissue into thermogenically active “brown-like” adipocytes and enhancing energy expenditure (81). Concurrently, studies in rats have shown that taurine upregulates peroxisome proliferator-activated receptor α (PPARα), thereby promoting transcription of genes involved in hepatic fatty acid oxidation, including acyl-CoA oxidase and carnitine palmitoyltransferase 1A, and accelerating lipid utilization for energy production (82). In contrast, taurine inhibits *de novo* lipogenesis by suppressing the activity of key lipogenic transcription factors, such as sterol regulatory element-binding protein-1c (SREBP-1c) and liver X receptor *α* (LXRα), thereby reducing hepatic lipid accumulation, as shown in cell culture and rodent studies (83, 84).

Insulin resistance represents a central pathological driver of lipid metabolic disorders. Taurine ameliorates obesity-associated chronic low-grade inflammation and enhances insulin signaling through its antioxidant and anti-inflammatory properties, as demonstrated in rodent models (8, 85). In genetically obese mouse models, taurine supplementation improves glucose homeostasis, preserves pancreatic *β*-cell function, modulates islet hormone secretion profiles, and mitigates hyperglycemia and hyperinsulinemia (85). Improved insulin sensitivity subsequently suppresses excessive adipose tissue lipolysis, reduces hepatic influx of free fatty acids, and decreases hepatic synthesis and secretion of very-low-density lipoproteins (VLDL), thereby establishing a beneficial feedback loop in lipid metabolic regulation (86, 87).

Recent evidence from mouse adipocyte studies further indicates that taurine promotes lipid turnover through autophagy-dependent mechanisms. Specifically, taurine induces nuclear translocation of transcription factor EB (TFEB) by inhibiting extracellular signal-regulated kinase 1/2 (ERK1/2) phosphorylation, leading to upregulation of autophagy-lysosome pathway-related genes and enhancement of adipocyte autophagic flux (88). This mechanism facilitates intracellular lipid droplet degradation and the clearance of dysfunctional organelles, further reinforcing taurine’s protective effects against obesity, insulin resistance, and chronic inflammation. Direct evidence regarding taurine’s role in canine lipid metabolism remains limited. A study in growing puppies suggested that taurine-containing neuroactive nutritional supplements can influence body fat accumulation and lipid oxidation metabolism (89), but further research is warranted to elucidate the species-specific functions and optimal dietary supplementation levels of taurine in the prevention and management of canine obesity and metabolic syndrome.

## 4 Key factors affecting canine taurine metabolism

Substantial interbreed differences exist in taurine biosynthetic capacity, transport efficiency, and metabolic regulation among dogs, which constitute a key genetic basis underlying variability in taurine deficiency risk. For instance, large-breed dogs exhibit significantly lower taurine biosynthesis rates than small-breed dogs (13). Certain breeds, including Doberman Pinschers and American Cocker Spaniels, display heightened sensitivity to taurine deficiency, and the incidence of DCM in these breeds is closely associated with disordered taurine metabolism (2, 30). Infantile dilated cardiomyopathy (IDCM) in Portuguese Water Dogs represents an autosomal recessive disorder, in which affected puppies exhibit reduced plasma taurine concentrations prior to the onset of clinical manifestations, providing direct evidence for the regulatory influence of genetic factors on taurine metabolism (90). These breed-specific differences may arise from genetic polymorphisms in taurine biosynthetic enzymes (e.g., CSAD) and from variation in the expression or activity of taurine transporters (TauT/SLC6A6), although the precise molecular mechanisms remain to be fully elucidated.

Body size represents an additional determinant of taurine requirements in dogs. Large-breed dogs possess a relatively higher body surface area–to–metabolic rate ratio and comparatively lower endogenous taurine synthesis capacity, resulting in substantially greater dietary taurine requirements than those of small-breed dogs (13). Taurine requirements also vary dynamically across physiological life stages. Puppies undergo rapid growth and organ development, particularly of the nervous, cardiovascular, and reproductive systems, processes that are highly dependent on taurine availability, while endogenous synthesis capacity remains immature, necessitating sufficient dietary supply. During pregnancy and lactation, taurine requirements in bitches increase markedly to support maternal homeostasis and to meet fetal and neonatal demands via placental transfer and milk secretion; taurine deficiency during these periods may adversely affect offspring growth and development. In elderly dogs, reduced taurine synthase activity, together with the frequent presence of chronic diseases such as cardiac and renal disorders, contributes to increased taurine consumption and loss, highlighting the need for targeted supplementation to maintain physiological homeostasis (14, 65).

Dietary composition constitutes the most critical exogenous determinant of taurine status in dogs, with its effects manifested through several interrelated mechanisms. Taurine is naturally abundant in animal-derived ingredients, including seafood, meat, and organ tissues, whereas plant-based raw materials such as grains and legumes contain negligible taurine levels (91, 92). Moreover, plant-based proteins are typically low in the precursor amino acids methionine and cysteine, which are essential for endogenous taurine synthesis. This factor, combined with the higher soluble fiber content often present in grain-free or plant-based diets—which increases fecal excretion of taurine-conjugated bile acids—can further exacerbate taurine depletion (24, 25). Consequently, grain-free diets predominantly formulated with plant proteins may predispose dogs to inadequate taurine intake, a finding consistent with the growing number of reported cases of diet-associated DCM linked to grain-free feeding practices in recent years (24). In addition, thermal processing and prolonged manufacturing procedures can substantially reduce taurine content in feed ingredients, as elevated temperatures accelerate taurine degradation and diminish dietary bioavailability (92). Dietary soluble fiber components, such as beet pulp, further reduce taurine reserves by increasing the excretion of taurine-conjugated bile acids in feces (25). High dietary levels of protein, fat, and soluble fiber exert synergistic effects in promoting bile acid loss, thereby increasing taurine requirements. Moreover, insufficient intake of sulfur-containing amino acids—methionine and cysteine, which serve as precursors for taurine biosynthesis—can limit endogenous taurine production and indirectly disrupt taurine homeostasis (9).

## 5 Discussion

This review systematically integrates current advances in the understanding of taurine in canine physiological homeostasis and nutritional regulation. Collectively, the evidence demonstrates that taurine, as a conditionally essential amino acid in dogs, exerts indispensable regulatory functions across multiple organ systems—including the myocardium, retina, central nervous system, reproductive system, and lipid metabolism—through core mechanisms such as calcium homeostasis regulation, mitochondrial functional maintenance, antioxidant defense, osmotic balance, and membrane stabilization. Taurine deficiency is closely associated with several canine diseases, notably dilated cardiomyopathy and retinal degenerative disorders, while breed-specific genetic susceptibility, body size, physiological stage, and dietary composition represent key determinants of taurine metabolic homeostasis. Given the generally limited endogenous taurine synthesis capacity in dogs, exogenous dietary supplementation is essential for maintaining physiological health, and precision taurine supplementation strategies tailored to individual needs are of critical importance for disease prevention and overall health maintenance.

The observed variation in taurine levels among dogs has identifiable biological underpinnings. Ko et al. (13) demonstrated that large-breed dogs have significantly lower endogenous taurine biosynthesis rates compared with small-breed dogs, with synthesis rate positively correlated with maintenance energy requirement per unit body weight. This finding suggests that taurine synthesis is metabolically costly, and larger dogs, which have lower metabolic rates per kilogram, may rely more heavily on dietary taurine acquisition. Beyond body size, breed-specific genetic factors also play a role. Certain breeds, such as Golden Retrievers and Newfoundlands, exhibit increased susceptibility to taurine deficiency even when fed diets apparently adequate in methionine and cysteine (2, 4). Evidence from controlled feeding studies further indicates that Newfoundlands have lower plasma cyst(e)ine concentrations and reduced *de novo* taurine synthesis compared with Beagles (93), and breed differences in hepatic enzyme activities have been proposed as contributing factors (94). Additionally, emerging evidence suggests that gut microbiota composition may influence taurine status, as Golden Retrievers with lower taurine levels showed subtle shifts in intestinal microbiota (31). Collectively, these studies indicate that inter-individual and interbreed variability in taurine status reflects a combination of body size-related metabolic scaling, breed-specific genetic polymorphisms, and potentially microbiota-mediated influences.

It is important to note that, unless otherwise specified, the mechanistic pathways discussed in this review are derived primarily from studies in laboratory animals and other mammalian species; direct experimental confirmation in dogs remains limited for many of these processes. Accordingly, the present review aims to synthesize the current mechanistic framework while explicitly highlighting areas where canine-specific data are needed. Despite substantial progress in elucidating taurine’s roles in canine nutrition and physiology, several important knowledge gaps and methodological limitations must be acknowledged. First, a large proportion of the mechanistic understanding presented in this review is derived from studies in non-canine species, including rodents, humans, and other mammals. While these studies provide valuable mechanistic insights, direct experimental confirmation in dogs remains limited for many physiological processes. Future investigations should prioritize the generation of canine-specific data to validate these mechanistic pathways. Second, as highlighted by the foundational and recent veterinary literature on taurine-associated DCM, the distinction between genetic susceptibility and dietary factors is critical for accurate diagnosis and intervention. The interplay between breed-specific genetic background, dietary composition (particularly grain-free and high-plant-protein diets), and taurine homeostasis warrants further investigation through well-controlled feeding trials and genetic association studies.

A recent scientific exchange highlights the complexity of interpreting the relationship between diet and cardiac function in dogs. Morris et al. (95) conducted an 18 month randomized, double-blind prospective study in 60 healthy adult dogs, comparing four diets with different ingredient profiles, including grain-free formulations with potatoes and peas. The study found that whole blood and plasma taurine levels remained within normal reference ranges across all diet groups, and all dogs were considered clinically normal with respect to DCM. The authors concluded that cardiac function was supported in healthy adult dogs fed foods formulated with different carbohydrate sources, including grain-free options.

In response, Pion (96) raised concerns regarding the interpretation of the echocardiographic data. He argued that dogs fed a grain-inclusive diet containing pea protein and pea fiber exhibited statistically significant changes in left ventricular dimensions and systolic function indices, which he interpreted as indicators of reduced myocardial function. He further contended that equating the absence of overt DCM with an absence of harm may overlook subclinical cardiac changes.

Notably, the diet associated with echocardiographic changes in the Morris et al. (95) study was a grain-inclusive diet containing pea protein and pea fiber—not a grain-free diet. This observation complicates the simplistic narrative that “grain-free diets cause DCM” and suggests that dietary factors beyond grain content, such as legume inclusion and overall nutrient formulation, may be relevant. The contrasting interpretations between Pion (96) and Morris et al. (95) underscore a fundamental scientific question: what threshold of evidence should be required to infer potential harm from dietary intervention? Pion (96) emphasizes statistically significant changes in echocardiographic parameters as potential early indicators of subclinical myocardial dysfunction, whereas Morris et al. (95) prioritizes clinical normalcy within established reference ranges as the benchmark for safety. Both positions have merit within their respective evidentiary frameworks, and resolving this tension will require further research specifically designed to address these methodological considerations.

From the perspective of different stakeholders, the implications of this debate vary. For veterinary clinicians, the exchange underscores the importance of individualized patient assessment rather than blanket dietary recommendations, and highlights the need to monitor both taurine levels and cardiac function in at-risk breeds. For canine nutritionists, the debate reinforces the principle that dietary formulation—including protein source, sulfur amino acid content, and fiber composition—may influence taurine homeostasis through multiple pathways. For dog owners and trainers, the current evidence suggests that grain-free does not equate to inherently dangerous, but breed predisposition, individual health status, and regular veterinary monitoring remain important considerations. For researchers, this debate highlights several priorities for future studies: (1) prospective studies specifically enrolling breeds known to be at higher risk for taurine deficiency; (2) inclusion of more sensitive cardiac biomarkers; (3) systematic variation of dietary protein sources, sulfur amino acid content, and fiber levels; and (4) investigation of the role of gut microbiota in taurine metabolism.

A comparison between cats and dogs offers additional insights into taurine physiology that can inform the canine debate. Cats are obligate carnivores with very limited capacity for endogenous taurine synthesis, due to low hepatic activity of cysteine dioxygenase and cysteine sulfinate decarboxylase (94). Consequently, cats must obtain taurine exclusively from their diet, and taurine deficiency in cats leads to well-characterized dilated cardiomyopathy and central retinal degeneration. The discovery by Pion et al. (97) that taurine deficiency was responsible for most cases of feline DCM led to mandatory taurine supplementation in commercial cat foods, dramatically reducing the incidence of the disease. Dogs, in contrast, possess a functional taurine biosynthetic pathway, albeit with significant breed and individual variability. A key metabolic difference is that cats conjugate bile acids exclusively with taurine, whereas dogs can conjugate bile acids with either taurine or glycine, providing greater metabolic flexibility when taurine availability is limited. As summarized by Li and Wu (94), compared with dogs, cats have greater endogenous nitrogen losses and higher dietary requirements for many amino acids (e.g., arginine, taurine, cysteine, and tyrosine), and are less sensitive to amino acid imbalances and antagonisms. Thus, cats have an absolute dietary requirement for taurine, while dogs have a conditional requirement that depends on breed, body size, diet composition, physiological state, and potentially gut microbiota composition. The feline experience provides an important lesson: it took decades of research to fully establish the causal link between taurine deficiency and DCM in cats, and the canine debate may similarly require long-term, well-controlled studies before definitive conclusions can be drawn.

Future studies should prioritize the identification of polymorphisms in taurine metabolism–related genes, such as CSAD and TauT, and the development of genotype–phenotype association models linking genetic markers with taurine requirements, thereby providing a molecular basis for breed-specific nutritional strategies. Moreover, while current research has predominantly focused on dilated cardiomyopathy, the mechanistic roles and preventive potential of taurine in other prevalent canine disorders—including cognitive dysfunction, diabetes mellitus, renal disease, and ophthalmic conditions—warrant systematic investigation. The synergistic or antagonistic interactions between taurine and other dietary components, such as sulfur-containing amino acids, antioxidants, and fatty acids, as well as their collective effects on metabolic homeostasis, remain incompletely characterized and require targeted interaction studies. In addition, emerging dietary formulations, including grain-free and high plant-protein diets, necessitate long-term feeding trials to assess their impacts on taurine homeostasis and associated health risks, thereby facilitating the establishment of evidence-based formulation standards. The ongoing scientific debate regarding the long-term feeding study by Morris et al. (95) and the critique by Pion (96)—along with the authors’ response (98)—highlights the need for further investigation in this area. Large-scale, long-term clinical trials are also needed to validate the efficacy of taurine supplementation in alleviating anxiety-related behaviors, enhancing working performance, and delaying age-associated functional decline, as well as to define optimal supplementation dosages, timing, and delivery methods.

The application of taurine in canine precision nutrition holds substantial practical value. In companion animal nutrition, individualized taurine supplementation strategies based on breed, body size, physiological stage, and health status can optimize diet formulations and reduce the risk of metabolic diseases. In working dog management, targeted taurine nutritional interventions have the potential to enhance physical endurance, cognitive performance, and emotional stability, thereby improving operational efficiency. Furthermore, in canine-assisted reproductive technologies, taurine serves as a key additive in sperm cryopreservation media, contributing to improved reproductive outcomes. With continued advances in research, the application of taurine in canine health management is expected to become increasingly precise and systematic, providing a robust scientific foundation for the advancement of canine nutritional science and comprehensive health protection frameworks.

## References

[1] Sanderson SL Gross KL Ogburn PN Calvert C Jacobs G Lowry SR . Effects of dietary fat and L-carnitine on plasma and whole blood taurine concentrations and cardiac function in healthy dogs fed protein-restricted diets. Am J Vet Res. (2001) 62:1616–23. doi: 10.2460/ajvr.2001.62.1616, 11592329

[2] Bélanger MC Ouellet M Queney G Moreau M. Taurine-deficient dilated cardiomyopathy in a family of golden retrievers. J Am Anim Hosp Assoc. (2005) 41:284–91. doi: 10.5326/0410284, 16141179

[3] Fascetti AJ Reed JR Rogers QR Backus RC. Taurine deficiency in dogs with dilated cardiomyopathy: 12 cases (1997–2001). J Am Vet Med Assoc. (2003) 223:1137–41. doi: 10.2460/javma.2003.223.1137, 14584743

[4] Backus RC Cohen G Pion PD Good KL Rogers QR Fascetti AJ. Taurine deficiency in Newfoundlands fed commercially available complete and balanced diets. J Am Vet Med Assoc. (2003) 223:1130–6. doi: 10.2460/javma.2003.223.1130, 14584742

[5] Duan H Song W Guo J Yan W. Taurine: a source and application for the relief of visual fatigue. Nutrients. (2023) 15:1843. doi: 10.3390/nu15081843, 37111062 PMC10142897

[6] Cruz GB Vasquez MA Cabañas E Joseph JN Skeen JC Lynch KP . Developmental lead exposure in rats causes sex-dependent changes in neurobiological and anxiety-like behaviors that are improved by taurine co-treatment. Adv Exp Med Biol. (2022) 1370:461–79. doi: 10.1007/978-3-030-93337-1_43, 35882819

[7] Li Y Peng Q Shang J Dong W Wu S Guo X . The role of taurine in male reproduction: physiology, pathology and toxicology. Front Endocrinol. (2023) 14:1017886. doi: 10.3389/fendo.2023.1017886, 36742382 PMC9889556

[8] Kp AD Martin A. Recent insights into the molecular regulators and mechanisms of taurine to modulate lipid metabolism: a review. Crit Rev Food Sci. (2023) 63:6005–17. doi: 10.1080/10408398.2022.2026873, 35040723

[9] Yang J Wu G Feng Y Sun C Lin S Hu J. CSD mRNA expression in rat testis and the effect of taurine on testosterone secretion. Amino Acids. (2010) 39:155–60. doi: 10.1007/s00726-009-0388-7, 19921479

[10] Kubo Y Ishizuka S Ito T Yoneyama D Akanuma S Hosoya K. Involvement of TauT/SLC6A6 in taurine transport at the blood-testis barrier. Meta. (2022) 12:66. doi: 10.3390/metabo12010066, 35050188 PMC8782047

[11] Cao T Zhang W Wang Q Wang C Ma W Zhang C . Cancer SLC6A6-mediated taurine uptake transactivates immune checkpoint genes and induces exhaustion in CD8(+) T cells. Cell. (2024) 187:2288–2304.e27. doi: 10.1016/j.cell.2024.03.011, 38565142

[12] Brosnan JT Brosnan ME. The sulfur-containing amino acids: an overview. J Nutr. (2006) 136:1636S–40S. doi: 10.1093/jn/136.6.1636S, 16702333

[13] Ko KS Backus RC Berg JR Lame MW Rogers QR. Differences in taurine synthesis rate among dogs relate to differences in their maintenance energy requirement. J Nutr. (2007) 137:1171–5. doi: 10.1093/jn/137.5.1171, 17449577

[14] Kriström K Häggström J Fascetti AJ Ström L Dirven M Yu J . The association between taurine concentrations and dog characteristics, clinical variables, and diet in English cocker spaniels: the canine taURinE (CURE) project. J Vet Intern Med. (2024) 38:2620–32. doi: 10.1111/jvim.17150, 39136304 PMC11423459

[15] Lee JY Jung DW Park HA Kim SJ Chung JH Moon CK . Effect of taurine on biliary excretion and metabolism of acetaminophen in male hamsters. Biol Pharm Bull. (2004) 27:1792–6. doi: 10.1248/bpb.27.1792, 15516725

[16] McCauley SR Clark SD Leach SB Quest BW Streeter RM. Evaluation of taurine and carnitine concentrations in whole blood, plasma, skeletal muscle and cardiac muscle in dogs. J Anim Physiol Anim Nutr (Berl). (2024) 108:999–1015. doi: 10.1111/jpn.13946, 38432690

[17] Schaffer SW Shimada-Takaura K Jong CJ Ito T Takahashi K. Impaired energy metabolism of the taurine-deficient heart. Amino Acids. (2016) 48:549–58. doi: 10.1007/s00726-015-2110-2, 26475290

[18] Santulli G Kansakar U Varzideh F Mone P Jankauskas SS Lombardi A. Functional role of taurine in aging and cardiovascular health: an updated overview. Nutrients. (2023) 15:4236. doi: 10.3390/nu15194236, 37836520 PMC10574552

[19] Oja SS Saransaari P. Taurine and the brain. Adv Exp Med Biol. (2022) 1370:325–31. doi: 10.1007/978-3-030-93337-1_31, 35882807

[20] Rinaldi A Cippà PE Nemazanyy I Anglicheau D Pallet N. Taurine deficiency is a hallmark of injured kidney allografts. Transplantation. (2024) 108:e218–28. doi: 10.1097/TP.0000000000004987, 39167563

[21] Ito T Murakami S. Taurine deficiency associated with dilated cardiomyopathy and aging. J Pharmacol Sci. (2024) 154:175–81. doi: 10.1016/j.jphs.2023.12.006, 38395518

[22] Chen Y Yi S Wang Q Li Y Lin S Liang S. Taurine supplementation alleviates asthma airway inflammation aggravated by HOCl exposure. J Hazard Mater. (2025) 490:137796. doi: 10.1016/j.jhazmat.2025.137796, 40058197

[23] Wu G San J Pang H Du Y Li W Zhou X . Taurine attenuates AFB1-induced liver injury by alleviating oxidative stress and regulating mitochondria-mediated apoptosis. Toxicon. (2022) 215:17–27. doi: 10.1016/j.toxicon.2022.06.003, 35688267

[24] Pezzali JG Shoveller AK Ellis J. Examining the effects of diet composition, soluble fiber, and species on total fecal excretion of bile acids: a meta-analysis. Front Vet Sci. (2021) 8:748803. doi: 10.3389/fvets.2021.748803, 34692814 PMC8529021

[25] Ko KS Fascetti AJ. Dietary beet pulp decreases taurine status in dogs fed low protein diet. J Anim Sci Technol. (2016) 58:29. doi: 10.1186/s40781-016-0112-6, 27489723 PMC4971673

[26] Liu M Zhang L Zhu K Guo H Zhu T Liu B . Taurine alleviates endoplasmic reticulum stress, oxidative stress, apoptosis, and glycogen accumulation induced by high glucose in the muscle cells of golden pompano (*Trachinotus ovatus*). Mar Life Sci Technol. (2025) 7:820–35. doi: 10.1007/s42995-025-00324-7, 41322274 PMC12662973

[27] Gray K Alexander LG Staunton R Colyer A Watson A Fascetti AJ. The effect of 48-hour fasting on taurine status in healthy adult dogs. J Anim Physiol Anim Nutr (Berl). (2016) 100:532–6. doi: 10.1111/jpn.12378, 26250395

[28] Delaney SJ Kass PH Rogers QR Fascetti AJ. Plasma and whole blood taurine in normal dogs of varying size fed commercially prepared food. J Anim Physiol Anim Nutr (Berl). (2003) 87:236–44. doi: 10.1046/j.1439-0396.2003.00433.x, 12752830

[29] UDAAL. (2023) UC Davis veterinary medicine amino acid lab. Available online at: https://www.vetmed.ucdavis.edu/labs/amino-acid-laboratory (Accessed September 11, 2025)

[30] Wess G Schulze A Butz V Simak J Killich M Keller LJM . Prevalence of dilated cardiomyopathy in Doberman pinschers in various age groups. J Vet Intern Med. (2010) 24:533–8. doi: 10.1111/j.1939-1676.2010.0479.x, 20202106

[31] Dametti MR Bagardi M Ghilardi S Minozzi G Polli M Brambilla PG . Preliminary analysis of intestinal microbiota in golden retrievers prone to dilated cardiomyopathy due to taurine deficiency. Vet Sci. (2025) 12:1120. doi: 10.3390/vetsci12121120, 41472100 PMC12737569

[32] Freid KJ Freeman LM Rush JE Cunningham SM Davis MS Karlin ET . Retrospective study of dilated cardiomyopathy in dogs. J Vet Intern Med. (2021) 35:58–67. doi: 10.1111/jvim.15972, 33345431 PMC7848368

[33] Kittleson MD Keene B Pion PD Loyer CG. Results of the multicenter spaniel trial (MUST): taurine- and carnitine-responsive dilated cardiomyopathy in American cocker spaniels with decreased plasma taurine concentration. J Vet Intern Med. (1997) 11:204–11. doi: 10.1111/j.1939-1676.1997.tb00092.x, 9298474

[34] Kaplan JL Stern JA Fascetti AJ Larsen JA Skolnik H Peddle GD . Taurine deficiency and dilated cardiomyopathy in golden retrievers fed commercial diets. PLoS One. (2018) 13:e0209112. doi: 10.1371/journal.pone.0209112, 30543707 PMC6292607

[35] Mornard L Brasileiro ACM Marcondes-Santos M. Role of diet as a predisposing factor for dilated cardiomyopathy in dogs: a narrative review. Vet Sci. (2025) 12:1106. doi: 10.3390/vetsci12111106, 41295744 PMC12656978

[36] Henry EF MacCormack TJ. Taurine protects cardiac contractility in killifish, *Fundulus heteroclitus*, by enhancing sarcoplasmic reticular Ca^2+^ cycling. J Comp Physiol B. (2018) 188:89–99. doi: 10.1007/s00360-017-1107-4, 28536755

[37] Ramila KC Jong CJ Pastukh V Ito T Azuma J Schaffer SW. Role of protein phosphorylation in excitation-contraction coupling in taurine deficient hearts. Am J Physiol Heart Circ Physiol. (2015) 308:H232–9. doi: 10.1152/ajpheart.00497.2014, 25437920

[38] Novotny MJ Hogan PM Paley DM Adams HR. Systolic and diastolic dysfunction of the left ventricle induced by dietary taurine deficiency in cats. Am J Phys. (1991) 261:H121–7. doi: 10.1152/ajpheart.1991.261.1.H121, 1858911

[39] Suzuki T Suzuki T Wada T Saigo K Watanabe K. Taurine as a constituent of mitochondrial tRNAs: new insights into the functions of taurine and human mitochondrial diseases. EMBO J. (2002) 21:6581–9. doi: 10.1093/emboj/cdf656, 12456664 PMC136959

[40] Schaffer SW Azuma J Mozaffari M. Role of antioxidant activity of taurine in diabetes. Can J Physiol Pharmacol. (2009) 87:91–9. doi: 10.1139/Y08-110, 19234572

[41] Kashio A Yamada C Yasuhara K Kamogashira T Someya S Yamasoba T. Taurine, coenzyme Q_10_, and hydrogen water prevents germanium dioxide-induced mitochondrial dysfunction and associated sensorineural hearing loss in mouse. Hear Res. (2023) 428:108678. doi: 10.1016/j.heares.2022.108678, 36577362 PMC11466212

[42] Mahmoud MM El-Batran SA Hegazy R El-Sayed WM. Taurine and enzymatically modified isoquercitrin protected against methotrexate-induced deteriorations in the conductivity and rhythmicity of the heart in rats: antioxidant, anti-inflammatory, and histological architecture approach. J Appl Toxicol. (2024) 44:1924–35. doi: 10.1002/jat.4682, 39135265

[43] Hamaguchi T Azuma J Schaffer S. Interaction of taurine with methionine: inhibition of myocardial phospholipid methyltransferase. J Cardiovasc Pharmacol. (1991) 18:224–30. doi: 10.1097/00005344-199108000-00008, 1717783

[44] Brethel S Locker S Girens R Rivera P Meurs K Adin D. The effect of taurine supplementation on the renin-angiotensin-aldosterone system of dogs with congestive heart failure. Sci Rep. (2023) 13:10700. doi: 10.1038/s41598-023-37978-1, 37400490 PMC10318075

[45] Ahmadian M Dabidi Roshan V Ashourpore E. Taurine supplementation improves functional capacity, myocardial oxygen consumption, and electrical activity in heart failure. J Diet Suppl. (2017) 14:422–32. doi: 10.1080/19390211.2016.1267059, 28118062

[46] Warskulat U Flögel U Jacoby C Hartwig H Thewissen M Merx MW . Taurine transporter knockout depletes muscle taurine levels and results in severe skeletal muscle impairment but leaves cardiac function uncompromised. FASEB J. (2004) 18:577–9. doi: 10.1096/fj.03-0496fje, 14734644

[47] Yatabe Y Miyakawa S Miyazaki T Matsuzaki Y Ochiai N. Effects of taurine administration in rat skeletal muscles on exercise. J Orthop Sci. (2003) 8:415–9. doi: 10.1007/s10776-002-0636-1, 12768487

[48] Bakker AJ Berg HM. Effect of taurine on sarcoplasmic reticulum function and force in skinned fast-twitch skeletal muscle fibres of the rat. J Physiol. (2002) 538:185–94. doi: 10.1113/jphysiol.2001.012872, 11773327 PMC2290020

[49] Sun B Maruta H Ma Y Yamashita H. Taurine stimulates AMP-activated protein kinase and modulates the skeletal muscle functions in rats via the induction of intracellular calcium influx. Int J Mol Sci. (2023) 24:4125. doi: 10.3390/ijms24044125, 36835534 PMC9962205

[50] Matsuzaki Y Miyazaki T Miyakawa S Bouscarel B Ikegami T Tanaka N. Decreased taurine concentration in skeletal muscles after exercise for various durations. Med Sci Sports Exerc. (2002) 34:793–7. doi: 10.1097/00005768-200205000-00011, 11984297

[51] Seo H Park S Song M. Diabetic retinopathy (DR): mechanisms, current therapies, and emerging strategies. Cells. (2025) 14:376. doi: 10.3390/cells14050376, 40072104 PMC11898816

[52] Shao M Wan Y Li Y Ma Y Ren J Li S . Association of oxidative stress biomarkers with primary congenital glaucoma. PLoS One. (2025) 20:e328663. doi: 10.1371/journal.pone.0328663, 40802678 PMC12348995

[53] Nishimura Y Hara H Kondo M Hong S Matsugi T. Oxidative stress in retinal diseases. Oxidative Med Cell Longev. (2017) 2017:4076518. doi: 10.1155/2017/4076518, 28424744 PMC5382422

[54] Madl JE McIlnay TR Powell CC Gionfriddo JR. Depletion of taurine and glutamate from damaged photoreceptors in the retinas of dogs with primary glaucoma. Am J Vet Res. (2005) 66:791–9. doi: 10.2460/ajvr.2005.66.791, 15934606

[55] Kim C Cha Y. Taurine chloramine produced from taurine under inflammation provides anti-inflammatory and cytoprotective effects. Amino Acids. (2014) 46:89–100. doi: 10.1007/s00726-013-1545-6, 23933994

[56] Bai H Li T Yu Y Zhou N Kou H Guo Y . Cytoprotective effects of taurine on heat-induced bovine mammary epithelial cells in vitro. Cells. (2021) 10:258. doi: 10.3390/cells10020258, 33525569 PMC7912084

[57] Froger N Cadetti L Lorach H Martins J Bemelmans A Dubus E . Taurine provides neuroprotection against retinal ganglion cell degeneration. PLoS One. (2012) 7:e42017. doi: 10.1371/journal.pone.0042017, 23115615 PMC3480351

[58] Liu C He P Guo Y Tian Q Wang J Wang G . Taurine attenuates neuronal ferroptosis by regulating GABA_B_/AKT/GSK3β/β-catenin pathway after subarachnoid hemorrhage. Free Radic Biol Med. (2022) 193:795–807. doi: 10.1016/j.freeradbiomed.2022.11.003, 36402441

[59] Neuwirth LS Emenike BU Cruz GB Cabañas E Vasquez MA Joseph JN . Taurine-derived compounds produce anxiolytic effects in rats following developmental lead exposure. Adv Exp Med Biol. (2022) 1370:445–60. doi: 10.1007/978-3-030-93337-1_42, 35882818

[60] Neuwirth LS Emenike BU Barrera ED Hameed N Rubi S Dacius TFJ . Assessing the anxiolytic properties of taurine-derived compounds in rats following developmental lead exposure: a neurodevelopmental and behavioral pharmacological pilot study. Adv Exp Med Biol. (2019) 1155:801–19. doi: 10.1007/978-981-13-8023-5_69 31468449

[61] Neuwirth LS Kim Y Barrerra ED Jo C Chrisphonte J Hameed N . Early neurodevelopmental exposure to low lead levels induces fronto-executive dysfunctions that are recovered by taurine co-treatment in the rat attention set-shift test: implications for taurine as a psychopharmacotherapy against neurotoxicants. Adv Exp Med Biol. (2019) 1155:821–46. doi: 10.1007/978-981-13-8023-5_70 31468450

[62] Zhu Y Wang R Fan Z Luo D Cai G Li X . Taurine alleviates chronic social defeat stress-induced depression by protecting cortical neurons from dendritic spine loss. Cell Mol Neurobiol. (2023) 43:827–40. doi: 10.1007/s10571-022-01218-3, 35435537 PMC9958166

[63] Mathis S Schoolfield S Gross P Gruen M Dorman DC. A systematic review of the efficacy of compression wraps as an anxiolytic in domesticated dogs. Animals (Basel). (2024) 14:3445. doi: 10.3390/ani14233445, 39682410 PMC11639916

[64] Salonen M Sulkama S Mikkola S Puurunen J Hakanen E Tiira K . Prevalence, comorbidity, and breed differences in canine anxiety in 13,700 Finnish pet dogs. Sci Rep. (2020) 10:2962. doi: 10.1038/s41598-020-59837-z, 32139728 PMC7058607

[65] Wu G. Roles of nutrients in the brain development, cognitive function, and mood of dogs and cats. Adv Exp Med Biol. (2024) 1446:177–202. doi: 10.1007/978-3-031-54192-6_8, 38625529

[66] Martins-Bessa A Rocha A Mayenco-Aguirre A. Effects of taurine and hypotaurine supplementation and ionophore concentrations on post-thaw acrosome reaction of dog spermatozoa. Theriogenology. (2009) 71:248–53. doi: 10.1016/j.theriogenology.2008.07.006, 18774600

[67] Yang J Wu G Feng Y Lv Q Lin S Hu J. Effects of taurine on male reproduction in rats of different ages. J Biomed Sci. (2010) 17:S9. doi: 10.1186/1423-0127-17-S1-S9, 20804629 PMC2994374

[68] Ostapiv RD Humenyuk SL Manko VV. Activity and isozyme content of lactate dehydrogenase under long-term oral taurine administration to rats. Ukr Biochem J. (2015) 87:54–62. doi: 10.15407/ubj87.04.054, 26547964

[69] DasGupta M Kumaresan A Saraf KK Paul N Sajeevkumar T Karthikkeyan G . Deciphering metabolomic alterations in seminal plasma of crossbred (*Bos taurus* X *Bos indicus*) bulls through comparative deep metabolomic analysis. Andrologia. (2022) 54:e14253. doi: 10.1111/and.14253, 34549825

[70] Banday MN Lone FA Rasool F Rashid M Shikari A. Use of antioxidants reduce lipid peroxidation and improve quality of crossbred ram sperm during its cryopreservation. Cryobiology. (2017) 74:25–30. doi: 10.1016/j.cryobiol.2016.12.008, 28040489

[71] Shiva Shankar Reddy N Jagan Mohanarao G Atreja SK. Effects of adding taurine and trehalose to a tris-based egg yolk extender on buffalo (*Bubalus bubalis*) sperm quality following cryopreservation. Anim Reprod Sci. (2010) 119:183–90. doi: 10.1016/j.anireprosci.2010.01.012, 20197223

[72] Oyovwi MO Nwangwa EK Ben-Azu B Rotue RA Edesiri TP Emojevwe V . Prevention and reversal of chlorpromazine induced testicular dysfunction in rats by synergistic testicle-active flavonoids, taurine and coenzyme-10. Reprod Toxicol. (2021) 101:50–62. doi: 10.1016/j.reprotox.2021.01.013, 33548410

[73] Xiao J Bi C Yang M Chen C Zhao J Huang X . Taurine alleviates inflammation, oxidative stress, apoptosis, and uterus microbiota dysregulation of endometritis by inhibiting PI3K-AKT/MAPK/NF-κB pathways in mice. Animals (Basel). (2025) 15:3619. doi: 10.3390/ani15243619, 41463904 PMC12729743

[74] Ommati MM Sabouri S Retana-Marquez S Nategh Ahmadi H Arjmand A Alidaee S . Taurine improves sperm mitochondrial indices, blunts oxidative stress parameters, and enhances steroidogenesis and kinematics of sperm in lead-exposed mice. Reprod Sci. (2023) 30:1891–910. doi: 10.1007/s43032-022-01140-5, 36484981

[75] Lam NV Chen W Suruga K Nishimura N Goda T Oda H . Effects of taurine on mRNA levels of nuclear receptors and factors involved in cholesterol and bile acid homeostasis in mice. Adv Exp Med Biol. (2006) 583:193–202. doi: 10.1007/978-0-387-33504-9_20, 17153602

[76] Murakami S Kondo Y Toda Y Kitajima H Kameo K Sakono M . Effect of taurine on cholesterol metabolism in hamsters: up-regulation of low density lipoprotein (LDL) receptor by taurine. Life Sci. (2002) 70:2355–66. doi: 10.1016/s0024-3205(02)01507-2, 12150200

[77] Lam NV Chen W Suruga K Nishimura N Goda T Yokogoshi H. Enhancing effect of taurine on CYP7A1 mRNA expression in Hep G2 cells. Amino Acids. (2006) 30:43–8. doi: 10.1007/s00726-005-0244-3, 16151615

[78] Fukuda N Yoshitama A Sugita S Fujita M Murakami S. Dietary taurine reduces hepatic secretion of cholesteryl ester and enhances fatty acid oxidation in rats fed a high-cholesterol diet. J Nutr Sci Vitaminol. (2011) 57:144–9. doi: 10.3177/jnsv.57.144, 21697633

[79] Yamamoto K Yoshitama A Sakono M Nasu T Murakami S Fukuda N. Dietary taurine decreases hepatic secretion of cholesterol ester in rats fed a high-cholesterol diet. Pharmacology. (2000) 60:27–33. doi: 10.1159/000028343, 10629440

[80] Karikkakkavil Prakashan AD Muthukumar SP Martin A. Taurine activates SIRT1/AMPK/FOXO1 signaling pathways to favorably regulate lipid metabolism in C57BL6 obese mice. Mol Nutr Food Res. (2024) 68:e2200660. doi: 10.1002/mnfr.202200660, 38549461

[81] Guo Y Li B Peng W Guo L Tang Q. Taurine-mediated browning of white adipose tissue is involved in its anti-obesity effect in mice. J Biol Chem. (2019) 294:15014–24. doi: 10.1074/jbc.RA119.009936, 31427436 PMC6791308

[82] Bonfleur ML Borck PC Ribeiro RA Caetano LC Soares GM Carneiro EM . Improvement in the expression of hepatic genes involved in fatty acid metabolism in obese rats supplemented with taurine. Life Sci. (2015) 135:15–21. doi: 10.1016/j.lfs.2015.05.019, 26092479

[83] Chen W Guo J Zhang Y Zhang J. The beneficial effects of taurine in preventing metabolic syndrome. Food Funct. (2016) 7:1849–63. doi: 10.1039/c5fo01295c, 26918249

[84] Lu Z He X Ma B Zhang L Li J Jiang Y . Dietary taurine supplementation decreases fat synthesis by suppressing the liver X receptor α pathway and alleviates lipid accumulation in the liver of chronic heat-stressed broilers. J Sci Food Agric. (2019) 99:5631–7. doi: 10.1002/jsfa.9817, 31106428

[85] Santos-Silva JC Ribeiro RA Vettorazzi JF Irles E Rickli S Borck PC . Taurine supplementation ameliorates glucose homeostasis, prevents insulin and glucagon hypersecretion, and controls β, α, and δ-cell masses in genetic obese mice. Amino Acids. (2015) 47:1533–48. doi: 10.1007/s00726-015-1988-z, 25940922

[86] Zhao D Lv Q Yang J Wu G Liu M Yang Q . Taurine improves lipid metabolism and skeletal muscle sensitivity to insulin in rats fed with high sugar and high fat diet. Adv Exp Med Biol. (2019) 1155:133–46. doi: 10.1007/978-981-13-8023-5_12, 31468392

[87] Sun Q Wang J Wang H Yu H Wan K Ma F . Effect of long-term taurine supplementation on the lipid and glycaemic profile in adults with overweight or obesity: a systematic review and meta-analysis. Nutrients. (2024) 17:55. doi: 10.3390/nu17010055, 39796489 PMC11722866

[88] Kaneko H Kobayashi M Mizunoe Y Yoshida M Yasukawa H Hoshino S . Taurine is an amino acid with the ability to activate autophagy in adipocytes. Amino Acids. (2018) 50:527–35. doi: 10.1007/s00726-018-2550-6, 29523960

[89] Wang W Brooks M Gardner C Milgram N. Effect of neuroactive nutritional supplementation on body weight and composition in growing puppies. J Nutr Sci. (2017) 6:e56. doi: 10.1017/jns.2017.57, 29209495 PMC5705811

[90] Alroy J Rush JE Sarkar S. Infantile dilated cardiomyopathy in Portuguese water dogs: correlation of the autosomal recessive trait with low plasma taurine at infancy. Amino Acids. (2005) 28:51–6. doi: 10.1007/s00726-004-0149-6, 15611846

[91] Ujong AE. Taurine as a functional ingredient: dietary sources, non-thermal extraction technologies, purification, potential health benefits and its applications. Food Chem. (2025) 503:147743. doi: 10.1016/j.foodchem.2025.147743, 41475256

[92] Spitze AR Wong DL Rogers QR Fascetti AJ. Taurine concentrations in animal feed ingredients; cooking influences taurine content. J Anim Physiol Anim Nutr (Berl). (2003) 87:251–62. doi: 10.1046/j.1439-0396.2003.00434.x, 12864905

[93] Backus RC Ko KS Fascetti AJ Kittleson MD Macdonald KA Maggs DJ . Low plasma taurine concentration in Newfoundland dogs is associated with low plasma methionine and cyst(e)ine concentrations and low taurine synthesis. J Nutr. (2006) 136:2525–33. doi: 10.1093/jn/136.10.2525, 16988121

[94] Li P Wu G. Amino acid nutrition and metabolism in domestic cats and dogs. J Anim Sci Biotechnol. (2023) 14:19. doi: 10.1186/s40104-022-00827-8, 36803865 PMC9942351

[95] Morris EM Stiers CA Hancock LB Gross KL. Different carbohydrate sources in dog foods supported overall health and cardiac function: an 18-mo prospective study in healthy adult dogs. J Anim Sci. (2025) 103:skaf225. doi: 10.1093/jas/skaf225, 40642821 PMC12408985

[96] Pion PD. Letter to editor regarding different carbohydrate sources in dog foods supported overall health and cardiac function: an 18-month prospective study in healthy adult dogs. J Anim Sci. (2026) 104:skag028. doi: 10.1093/jas/skag028, 41738070 PMC12972674

[97] Pion PD Kittleson MD Rogers QR Morris JG. Myocardial failure in cats associated with low plasma taurine: a reversible cardiomyopathy. Science. (1987) 237:764–8. doi: 10.1126/science.3616607, 3616607

[98] Morris EM Stiers CA Hancock LB Gross KL. Response to the letter to the editor from Pion et al. regarding “different carbohydrate sources in dog foods supported overall health and cardiac function”. J Anim Sci. (2026) 104:skag029. doi: 10.1093/jas/skag029, 41688882 PMC12972676

[99] Ontiveros ES Whelchel BD Yu J Kaplan JL Sharpe AN Fousse SL . Development of plasma and whole blood taurine reference ranges and identification of dietary features associated with taurine deficiency and dilated cardiomyopathy in golden retrievers: a prospective, observational study. PLoS One. (2020) 15:e0233206. doi: 10.1371/journal.pone.0233206, 32413894 PMC7228784

[100] Furlanello T Masti R Bertolini FM Ongaro V Zoia A Sanchez Del Pulgar J. Development and validation of a robust and straightforward LC-MS method for measuring taurine in whole blood and plasma of dogs and reference intervals calculation. Animals (Basel). (2024) 15:3. doi: 10.3390/ani15010003, 39794945 PMC11718811

[101] Kathrani A Allenspach K Fascetti AJ Larsen JA Hall EJ. Alterations in serum amino acid concentrations in dogs with protein-losing enteropathy. J Vet Intern Med. (2018) 32:1026–32. doi: 10.1111/jvim.15116, 29604114 PMC5980272

